# Daytime or Edge-of-Daytime Intra-Canopy Illumination Improves the Fruit Set of Bell Pepper at Passive Conditions in the Winter

**DOI:** 10.3390/plants11030424

**Published:** 2022-02-04

**Authors:** Vivekanand Tiwari, Itzhak Kamara, Kira Ratner, Yair Many, Victor Lukyanov, Carmit Ziv, Ziva Gilad, Itzhak Esquira, Dana Charuvi

**Affiliations:** 1 Institute of Plant Sciences, Agricultural Research Organization (ARO), Volcani Center, Rishon LeZion 7505101, Israel; vivekcas805@gmail.com (V.T.); itzahk@volcani.agri.gov.il (I.K.); kiratner@volcani.agri.gov.il (K.R.); yairm@volcani.agri.gov.il (Y.M.); 2 Institute of Soil, Water and Environmental Sciences, ARO, Volcani Center, Rishon LeZion 7505101, Israel; viclukyanov@gmail.com; 3 Institute Postharvest and Food Sciences, ARO, Volcani Center, Rishon LeZion 7505101, Israel; carmit.ziv@volcani.agri.gov.il; 4 Jordan Valley Research and Development Authority, Mobile Post 9190600, Israel; ziva@mop-bika.org.il; 5 Faculty of Sciences and Technology, Tel-Hai College, Upper Galilee, Kiryat Shmona 1220800, Israel; esquirai@gmail.com

**Keywords:** light-emitting diodes (LEDs), intra-canopy illumination, interlighting, bell pepper, photosynthesis, fruit set, daily light integral (DLI)

## Abstract

Optimal light conditions ensure the availability of sufficient photosynthetic assimilates for supporting the survival and growth of fruit organs in crops. One of the growing uses of light-emitting diodes (LEDs) in horticulture is intra-canopy illumination or LED-interlighting, providing supplemental light for intensively cultivated crops directly within their canopies. Originally developed and applied in environmentally controlled greenhouses in northern latitude countries, this technique is nowadays also being tested and studied in other regions of the world such as the Mediterranean region. In the present work, we applied intra-canopy illumination for bell pepper grown in passive high tunnels in the Jordan Valley using a commercial LED product providing cool-white light. The study included testing of daytime (‘LED-D’) and edge-of-daytime (‘LED-N’) illumination, as well as a detailed characterization of fruit set and fruit survival throughout the growth season. We found that both light regimes significantly improved the fruit set and survival during winter, with some benefit of LED-N illumination. Notably, we found that western-facing plants of illuminated sections had a higher contribution toward the increased winter fruit set and spring yield than that of illuminated eastern-facing plants. Greater plant height and fresh weight of western-facing plants of the illuminated sections support the yield results. The differences likely reflect higher photosynthetic assimilation of western-facing plants as compared to eastern-facing ones, due to the higher daily light integral and higher canopy temperature of the former. This study provides important implications for the use of intra-canopy lighting for crops grown at passive winter conditions and exemplifies the significance of geographical positioning, opening additional avenues of investigation for optimization of its use for improving fruit yield under variable conditions.

## 1. Introduction

Light is one of the most important factors for crop production. Light supplementation is a common practice in greenhouses, particularly during winter in northern latitude countries. Nonetheless, even in regions that do not ‘suffer’ from a severe lack of light, the crop canopy can be light-limited due to self-shading, its geographical position, or cultivation in wintertime. Over the past two decades or so, light-emitting diodes (LEDs) have been replacing the conventional (fluorescent; incandescent; high-pressure sodium; metal halide) lighting sources. Nowadays, the use of LEDs in horticulture is quite widespread, including in middle latitudes, and has numerous advantages over other types of lighting [1,2,3,4,5]. One of these is the possibility to illuminate plants directly within their canopies without resulting in heat-induced damage, termed ‘intra-canopy illumination’ or ‘interlighting’ [6]. LED interlighting has been mostly applied for high-wire intensive crops, such as tomato, pepper, and cucumber, grown in environmentally controlled greenhouses, in addition to, or as an alternative to, overhead illumination during winter [7,8]. Use of intra-canopy illumination for these crops, in which tall and dense canopies can result in excessive shading, has been shown to improve plant growth and fruit yield (number and/or size) as well as affect fruit quality [9,10,11]. Over the past few years, studies and testing of interlighting have spread to additional regions of the world [12,13,14].

Pepper (*Capsicum annuum*) belongs to the Solanaceae (nightshade) family and is one of the world’s most consumed fruit—in raw or cooked form, as well as processed into spices, condiments, or coloring agents [15]. Pepper fruit is an important source of vitamin A, -C, and -E, flavonoids, carotenoids, and additional antioxidant metabolites, highlighting its importance for human nutrition and health [16]. Bell pepper is cultivated as an annual crop all over the world, with long growth periods spanning different seasons. In countries with extremely cold winters and limited natural light, e.g., Canada, the Netherlands, or northern regions of the United States of America, bell pepper is grown in environmentally controlled greenhouses, with high-quality fruit harvest in spring and summer. In tropical, semi-arid climates such as Mexico, the crop is grown (for the most part) with minimal or without climate control. In other regions of the world, such as the Mediterranean basin, bell pepper is grown as a protected crop, with the advantage of being able to produce high quality fruit during winter.

Light limitation in winter is a major factor limiting fruit yield. Even in countries with mostly mild winters, the daily light integral (DLI) in winter is low [12] and temperatures fall below those that are optimal for growth and photosynthesis of bell pepper. Furthermore, the crop cultivation technique can also result in considerable shading. Growth in the “Spanish” trellis system has the benefits of higher yields of large fruit size, a lower rate of blossom-end rot, and 75% lower labor costs required for pruning, over the “V” system for bell pepper cultivation [17]. Nonetheless, a lack of pruning of branches or leaves reduces light penetration into the canopy, resulting in disadvantageous non-uniform light distribution and reduced photosynthesis [18].

Bell pepper grown under passive (protected) conditions is characterized by waves of fruit production, with variable time kinetics for fruit growth, development, and ripening along the growth season. The pattern of waves is determined by the environmental conditions, which affect the photosynthetic efficiency, as well as by the on-plant fruit load, together influencing source–sink relations and ultimately organ (flower bud, flower, young fruits) development and/or survival. Sweet pepper is generally characterized by high organ abortion rates, affected by various factors, as reviewed in [19], with light being a predominant one. Experiments in which bell pepper plants were subjected to low light by shading, or where adjacent leaves were removed, showed that these conditions correlated with reduced sugar accumulation in the flower, increasing flower abscission and reducing fruit set [20]. Additional studies also showed that source and sink strengths are major determinants of organ abortion in pepper [21,22], and that the fruit sink strength can affect the photosynthetic characteristics of proximal leaves [23]. Extending the photoperiod, up to a certain extent, was reported to increase pepper fruit yield [24,25]. Improving photosynthesis, by providing optimal light, CO_2_, and temperature conditions, would improve the source strength, reduce organ abortion [19], and support the development of more fruit, thus increasing the yield.

Various studies, conducted at different conditions, have shown that light supplementation of the pepper plant canopy can improve the yield. Increasing the photosynthetic photon flux at different heights of the pepper plant canopy (using HPS lamps at the time) resulted in a 23% increase in the total fruit yield [26]. Later studies demonstrated the improvement of pepper fruit yield using LED-interlighting applied in environmentally controlled greenhouses [10,27]. We recently reported that application of daytime intra-canopy LED illumination for bell pepper grown in passive tunnels in the Jordan Valley results in increased yield during the spring months, due to a higher number of fruit [12]. In a follow-up experiment, we found indications for a higher number of fruitlets in western-facing plants of the double-row beds that were illuminated, as compared to non-illuminated plants on the same side. On the other hand, there was no difference in the number of fruitlets in eastern-facing plants with or without illumination. In the current study, we applied intra-canopy illumination for bell pepper using a commercial product and aimed to (1) compare the effects of daytime- (‘LED-D’) vs. edge-of-daytime (‘LED-N’) illumination; and (2) characterize fruit set and fruit survival under the two illumination regimes for eastern- and western-facing plants, as compared to non-illuminated ones.

## 2. Materials and Methods

### 2.1. Plants and Growth Conditions

The study was carried out at the ‘Zvi’ R&D Experimental Station in the Jordan Valley (31°59′49.0″ N, 35°27′09.3″ E) from August 2019 to May 2020. Crop management followed the routine practices of the region. Prior to planting, soil sanitation was carried out by solar fumigation for 4 weeks, with streaming of metam sodium (40 mL m^−2^) into the soil via drip irrigation during the last week. Red bell pepper seedlings (*Capsicum annuum* L. cv. Cannon, Zeraim Gedera/Syngenta, Revadim, Israel) were planted on Aug. 20, 2019, in a high tunnel (10 m wide × 4 m high × 45 m long) in the local soil, a well-drained (EC < 2.0 dS m^−1^) clay (30%)–limestone (50%) marl soil. Planting was in double-row beds of width 0.8 m, and center-to-center distance between beds of 1.75 m, with an overall plant density of 2.9 m^−2^. Plant training applied the ‘Spanish’ trellis system, with lateral horizontal wires supporting the canopy vertically, and without pruning.

Drip irrigation (emitter flow rate 1.6 L h^−1^) was provided every 20 cm along each plant row, with a total of ~8000 m^3^ ha^−1^ per growth season (from planting in Aug. until May), similarly to commercial plots. Crop irrigation varied according to evapotranspiration calculated (Penman-Monteith FAO56, [28]) from the local meteorological data: ~40 m^3^ ha^−1^ d^−1^ from planting to mid-Dec., ~10 m^3^ ha^−1^ d^−1^ from mid-Dec. to the end of Feb., and 60–70 m^3^ ha^−1^ d^−1^ in March to May. Fertigation with N-P-K (6:3:9, ICL, Tel-Aviv, Israel) was provided at a concentration that varied between 1 to 1.5 L m^−3^ until Feb., and 0.5 L m^−3^ afterward. Fe (5 kg ha^−1^ of Sequestrene Fe 6%, Syngenta) and Mn (15 L ha^−1^ of Koratin-Mn 18 g L^−1^, ICL Israel) were provided 3 times, at the beginning of November, mid-December, and the beginning of March.

At the time of planting, the tunnel was covered by a 50-mesh insect-proof screen with a black shade net (40%) on top of it. On 19 September 2019, the shade net was removed, and on 17 November 2019, the mesh screen was replaced by a polyethylene sheet (Ginegar Plastic Products Ltd., Kibbutz Ginegar, Israel). To prevent fruit heat damage, on 16 March 2020, the plastic sheet was removed and the 50-mesh screen together with the black shade net (40%) were placed on top of the tunnel until the end of the experiment. The fruit yield was followed in the spring, with harvesting according to the commercial standard of picking at >60% red color. Both ‘class 1’ (export-quality fruit) and ‘class 2’ (for local market) were included in the spring yield.

### 2.2. Supplemental Intra-Canopy Illumination

**Figure 1:**
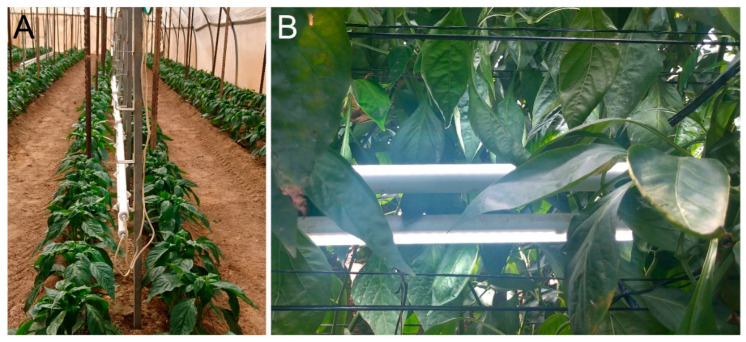
**Intra-canopy supplemental illumination.** (**A**) Installation of the ‘Bioled’ light fixtures at the center of the beds (September 2019, prior to start of illumination treatments). (**B**) Side-view of intra-canopy back-to-back LED illumination (picture acquired in March 2020).

**Figure 2:**
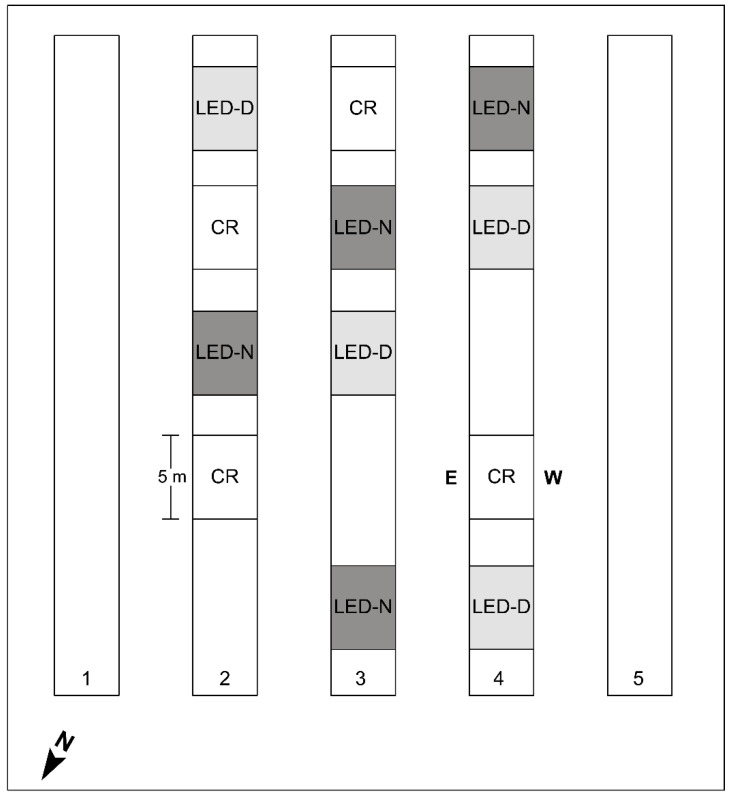
**Schematic map of the experimental tunnel.** The experiment was carried out in the three central double-row beds (2, 3, and 4) of the tunnel. Intra-canopy lighting was applied at the center of the beds between the two rows (Figure 1A), along 5 m-long sections. The illumination was applied either during daytime (‘LED-D’) or the two edges of daylight period (‘LED-N’), with non-illuminated sections of the same length as controls (‘CR’). Each treatment had four replicates. A spacing of at least 2 m was kept between sections. ‘E’ and ‘W’ denote the eastern- and western-facing rows, with regard to the results presented in further Figures and Tables. Note that beds and experimental sections are not drawn to scale.

The supplemental LED illumination was assembled from Crops IP67 tubes (Bioled Eco Light Systems Ltd., Tzova, Israel), providing cool-white (CW; 5700K) light at 32 W/m. CW was chosen as it was found to be preferable for bell pepper in our earlier study [12]. For simplicity, we refer to the LED tubes as ‘Bioled’ in the text. Two LED tubes affixed back-to-back were installed between the two adjacent rows of the beds (Figure 1). Two illumination regimes of 12 h were provided: daytime (‘LED-D’, 6:00 to 18:00) and edge-of-daytime (‘LED-N’, 4:00–10:00 and 16:00–22:00). The experimental setup encompassed four replicate sections (5.4-m-long each) for each of the two intra-canopy light treatments and for the non-illuminated control (Figure 2). The illumination period began 70 days after planting (28 October 2019), when the canopy height was ~1.5 m. Fixtures were installed at a height of 70–80 cm aboveground at the start of the illumination period, raised to 90–100 cm in the middle of December 2019, and raised again to 110–120 cm in the middle of March 2020.

Spectra and photosynthetic photon flux densities (PPFD) were recorded using a portable spectroradiometer (EPP2000C, StellarNet, Inc., Tampa, FL, USA) with a cosine-corrected head (Apogee Instruments Inc., Logan, UT, USA) and an LI-250A quantum sensor (LI-COR, Lincoln, NE, USA), respectively. Air temperature within the canopy was recorded using HOBO temperature data loggers (Onset Computer Corporation, Bourne, MA, USA) hung in proximity (10–15 cm) to the LED fixtures or at the same height in control sections.

### 2.3. Chlorophyll Content and Fluorescence

Measurements were conducted non-destructively on attached leaves of the inner canopy. Chlorophyll (Chl) content was assayed using an MC-100 Chl measurement system (Apogee, Chesapeake, VA, USA). Chl-*a* fluorescence emission was measured using a portable pulse-amplitude-modulated fluorometer (PAM-2000, Heinz Walz GmbH, Pfullingen, Germany) at its default setting designed to determine Fv/Fm (‘Da-2000’ program). In brief, leaves were subjected to dark adaptation for 20 min using dark leaf clips (DLC-8, Walz), and then initial Chl-*a* fluorescence (F0) and maximum Chl-*a* fluorescence in dark (Fm) were recorded after applying a saturating light pulse for 0.8 s. The Fv/Fm = [(Fm − F0)/Fm] values were calculated by the program and recorded.

### 2.4. Gas-Exchange Measurements

Gas-exchange measurements were conducted on attached leaves of the inner or outer canopy using a portable LCi photosynthesis system (ADC BioScientific Ltd., Hoddesdon, UK) with a clear top chamber at ambient conditions. For the inner canopy, leaves were sampled 10–15 cm above the upper LED fixtures, at a height of 110–120 cm above the ground, and at the same height in non-illuminated plots. For outer canopy measurements, leaves from the eastern- and western-facing canopy were probed in the morning or afternoon during peak photosynthetically active radiation (PAR) intensities on each side. Positioning of the LCi chamber was adjusted according to the leaf being measured (to keep the leaf attached) and to allow natural sunlight or light from the LEDs to reach the leaf.

### 2.5. Fruit Set Quantification

In each of the experimental replicate sections (four sections for each treatment) shown in Figure 2, ten plants were selected for the analysis: five in the eastern-facing row and five in the western-facing row of the same bed. Fruitlets were counted, labelled, and screened for survival on eleven dates throughout the season. Survival of fruit labelled on one date were assayed on the next fruitlet labelling date. On the last day of the experiment (7 May 2020), fruitlets remaining on the plants were counted.

### 2.6. Daily Light Integral (DLI) Recording

PAR (400–700 nm) was recorded using LI-190SB-L quantum sensors (LI-COR, Lincoln, NE, USA) installed at the eastern- and western-facing canopy (at a height of 1 m above ground), as well as above the canopy (height of 3 m) at 90 degrees. Data were recorded every ten minutes and logged by a Campbell system (CR10X—Campbell Scientific, Logan, UT, USA).

### 2.7. Statistical Analysis

Statistical analysis was performed using one-way analysis of variance (ANOVA), followed by Tukey’s multiple comparisons as a post hoc test. For comparisons of two groups, the Student’s *t*-test was used. The level of significance is provided in the figure legends/table.

## 3. Results

### 3.1. Intra-Canopy Illumination

**Figure 3:**
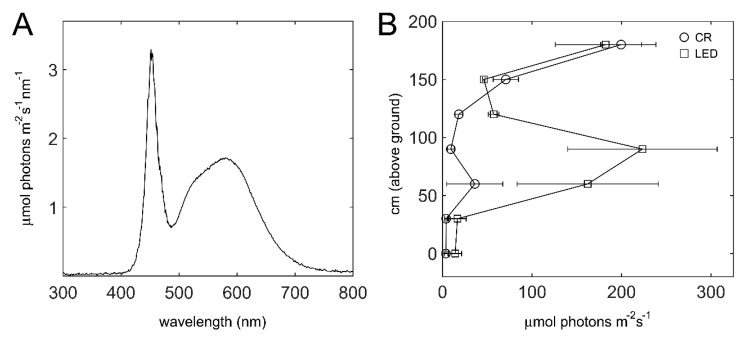
**Light spectrum and intensity within the canopy.** (**A**) Spectra of the cool-white ‘Bioled’ light fixtures. (**B**) Light intensity within the canopy in control vs. illuminated sections, recorded in December 2019. Intensities were measured with the light sensor directly below or above the fixtures at the indicated distances above the ground. Values shown are means ± SD of three control (CR) and three illuminated (LED) plots.

The ‘Bioled’ light fixtures utilized as intra-canopy illumination provided cool-white (CW) light (Figure 3A). The effect of the added illumination on the light intensity between the double-row beds was assessed when the canopy height was ~2 m. In control (‘CR’) non-illuminated sections, the light intensity of the inner canopy below 1.5 m was generally < 50 µmol photons m^−2^ s^−1^, and for the most part even <20 µmol photons m^−2^ s^−1^ (Figure 3B). In the illuminated sections, a region of almost 1 m in height, from 30 to 120 cm aboveground, exhibited significantly higher light intensities, reaching an average intensity of 225 µmol photons m^−2^ s^−1^ in proximity to the fixtures (Figure 3B). At a canopy height of 1.5 to 1.8 m, the light intensities in the CR and illuminated sections were similar, and the considerable higher intensity at 1.8 m is due to sunlight penetration at this height.

The effects of the supplemental illumination, provided by Bioled fixtures, on the photosynthetic parameters and gas-exchange activity of inner canopy leaves were characterized in LED-D plots (Table 1). The chlorophyll content (‘Chl’; measured non-destructively) was somewhat higher (~8%) in the illuminated leaves as compared to control ones. The CO_2_ assimilation rates (‘A’) in leaves of LED sections were ~3.3-fold higher than non-illuminated leaves. The stomatal conductance (‘Gs’) and transpiration rate (‘E’) were, respectively, 5.2- and 3.5-fold higher in illuminated leaves vs. control ones. As the temperature of the leaves probed for the gas-exchange recordings was the same in both control and LED, the higher Gs and E can be attributed to the supplemental light. The average light intensity, recorded during the measurements (‘PAR’), was ~3.7-fold higher in LED than in CR.

The effect of the illumination on air temperature within the canopy, in vicinity of the LED fixtures, was recorded along the season. The daily minimal (T-min) and maximal (T-max) air temperatures in the canopy of the three treatments are shown in Figure S1. Three examples for raw air temperature data on representative days depict how air temperature in the canopy is affected by operation of the illumination in LED-D and LED-N (Figure S2). In CR sections, T-min typically occurs between 04:00 and 07:00, and T-max between 12:00 and 15:00. The timing of T-max depends on the time of year and on whether the day is sunny (Figure S2A) or cloudy (Figure S2B). In LED-D sections, where the illumination operated from 06:00 to 18:00, the air temperature was higher by ~4.5 °C than the CR during the operation time. Accordingly, T-max was higher to a similar extent on most days in LED-D (Figure S1). In LED-N sections, higher air temperatures within the canopy were observed at the edges of daytime, in line with the operation times of the illumination for this treatment. As compared to CR, the air temperature within the canopy in LED-N was higher by 3.9 to 5.2 °C from 04:00 to 8:00 and by 4.2 to 5.6 °C from 16:00 to 22:00 (Figure S2). On some days, T-max in LED-N was higher than in CR and occurred around 10:00, just prior to the end of the first illumination period (Figure S2B,C). This is also evident in the whole season graph for T-max (Figure S1). Although LED-N operated from 04:00 to 10:00, the effect of the illumination on daily T-min was minor (Figures S1 and S2). The increased air temperature observed in LED treatments is mostly limited to the regions surrounding the light fixtures. Even though the increase in air temperature may not necessarily result in considerable increases in foliage temperature, it should still be kept in mind as a factor that can affect the plant physiology.

### 3.2. Supplemental Illumination Results in Increased Fruit Set in the Winter

We previously (2016–2018) found that using intra-canopy illumination in our experimental conditions increases the pepper fruit spring yield by ~30% [12]. In another experiment carried out with the Bioled CW fixtures used for daytime illumination (2018–2019), we quantified the fruit set accumulation during two months in the winter (Figure S3). We found that plants that were illuminated (at their inner canopy) had 46% more fruitlets than the control (whole ‘total’ bars). However, after assaying the fruitlet survival, illuminated plants remained with ~16% more fruit than control (colored part of ‘total’ bars). Notably, the increase in fruit number arose from western (W)-facing illuminated plants. These plants had 80% more fruitlets as compared to the W-facing control plants (whole ‘western’ bars). After assaying the fruit survival, there was 33% more fruit on the W-facing illuminated plants vs. control (colored part of bars). Conversely, the fruit set and fruit survival of the eastern-(‘E’) facing plants was nearly identical in illuminated and control plants. These earlier results provided the rationale for the current analysis of fruit set, as described below and in Section 3.3.

**Figure 4:**
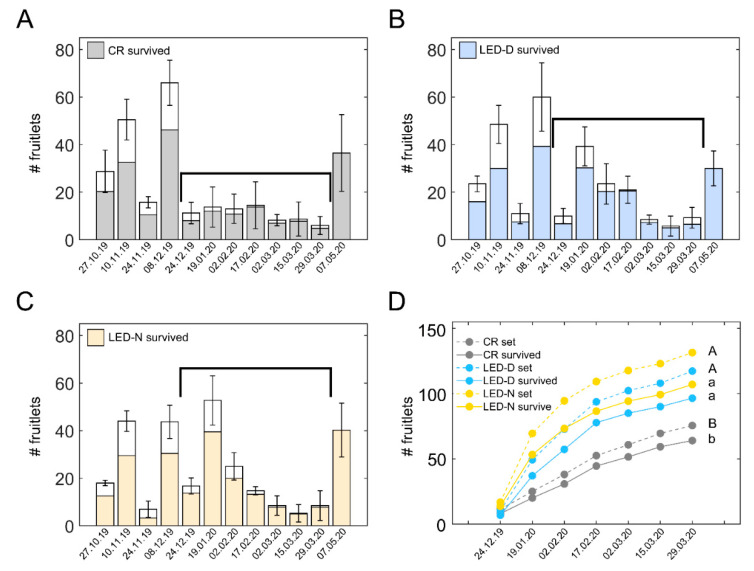
**Supplemental intra-canopy illumination increases fruit set and survival in the winter.** Fruitlets were labelled along the season in (**A**) control non-illuminated (CR) sections, (**B**) sections illuminated during daytime (LED-D), and (**C**) sections illuminated at the edge of day (LED-N). Whole bars denote the average number ± SD of fruitlets from four sections. In each section, the number of fruitlets was summed for ten plants: five in the eastern-facing row and five in the western-facing row of the same bed. Each section was from a different replicate in the experimental plot, see Figure 2) labelled on the noted dates. Colored portion of the bar shows the fraction of surviving fruits from the total. At the end of the experiment (07.05.20 bar), fruitlets remaining on the plants were counted without follow-up for survival. Black brackets denote the winter time period between the two big fruit set waves of control non-illuminated plants (**A**). (**D**) Cumulative number of fruitlets (dashed lines) and surviving fruit (solid lines) during the winter, corresponding to the period marked by the brackets in (**A**–**C**). Distinct upper- and lower-case letters denote statistically significant differences (*p* < 0.05) for the total number of labelled fruitlets and for surviving fruit, respectively.

To gain detailed insight into the fruit set behavior of illuminated vs. non-illuminated plants, in the current study, we followed the fruit set and survival in LED-D and LED-N light treatments and in the CR throughout the entire experiment (Figure 4). Panels A–C of this figure depict the average count of fruitlets and the surviving fraction from the four replicate sections of each treatment. In each section, the number of fruitlets were summed for ten plants (five from the E-facing row and five from the W-facing row; see Figure 2). Whole bars (means ± SD), including white and colored parts, show the average number of fruitlets counted on the indicated date. The colored parts of the bars indicate the surviving fruit, assayed two weeks later.

We were specifically interested in the winter period, during which an improved fruit set would lead to an increased yield in spring months. Examining the fruit set in CR sections, relatively low levels are evident between the end of December to the end of March (Figure 4A). This three-month period is marked by the black brackets in Figure 4A–C. Notably, during this period, plants from both LED-D (Figure 4B) and LED-N (Figure 4C) exhibited higher fruit set and survival, specifically during the coldest part of the winter (Figure S4). Figure 4D depicts the cumulative fruit set and fruit survival during the aforementioned time period. Fruit set (dashed lines) was considerably higher in both LED-D and LED-N, respectively, by 55% and 74%, as compared to the CR. Likewise, the number of surviving fruit (solid lines) in LED-D and LED-N were 51% and 67% higher than the CR.

### 3.3. Fruit Set and Survival Are Enhanced in Illuminated Western-Facing Plants

**Figure 5:**
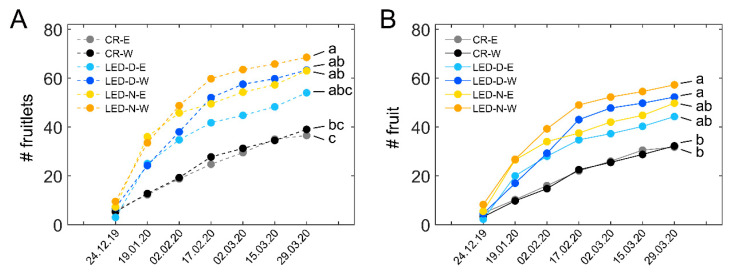
**Fruit set and survival in eastern- and western-facing plants.** (**A**) Cumulative number of fruitlets labelled during the winter in the eastern (E)- and western (W)-facing plants of control non-illuminated (CR) sections, and in the E- and W-facing sections illuminated during daytime (LED-D) or edge of day (LED-N). Values shown represent the average number ± SD of fruitlets from four E or W sections, each from a different replicate in the experimental plot, see Figure 2). For each E or W section, fruitlets were summed for five plants. (**B**) Cumulative surviving fruit from the E- and W-facing plants of the different treatments, corresponding to the fruitlets that were labelled (**A**). Distinct letters denote statistically significant differences (*p* < 0.05) between the six groups. Data shown in this figure are the same data shown in Figure 4D, separated to E and W.

Following the data we obtained in the earlier experiment for winter fruit set and survival in E- and W-facing plants (Figure S3), we also assessed the data shown in Figure 4D separately for E and W. Figure 5 shows the fruit set and survival in the three treatments of the study (CR, LED-D, LED-N) in E- and W-facing plants. For each treatment, E is shown by the lighter-colored lines and W by the darker-colored lines of the same shade. In illuminated sections of either LED-D or LED-N, the fruit set was higher in W-facing plants as compared to E-facing ones of the same treatment (Figure 5A). Nonetheless, in CR sections, the fruit set was nearly identical in E- and W-facing plants. The highest number of fruitlets was observed in LED-N-W plants (Figure 5A, orange line), 76% higher than CR-W. From the light treatments, LED-D-E had the lowest, but still considerably high, number of fruitlets, 48% higher than CR-E. The number of surviving fruit (Figure 5B) reflects that of the fruit set. Only W-facing plants, of both LED-D and LED-N, exhibited a significantly higher number of surviving fruit as compared to the CR. These were 78% and 62% higher for LED-N-W and LED-D-W, respectively. The trend for a higher number of fruit in LED-N vs. LED-D was observed in both fruit set and fruit survival, although the differences between the two were not statistically significant.

### 3.4. Daily Light Integral and Photosynthetic Activity of the Eastern- and Western-Facing Canopy

**Figure 6:**
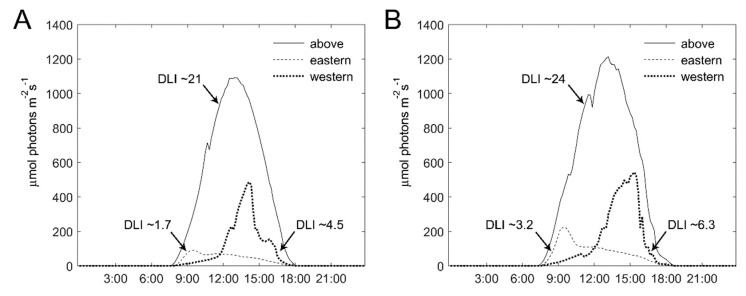
**Photosynthetically-active radiation of sunlight at the outer canopy of eastern- and western-facing plants.** Photosynthetically-active radiation (PAR; 400–700 nm) shown for two sunny days: (**A**) 6 January 2020 and (**B**) 17 February 2020, was recorded above the canopy (at a height of ~3 m), and at the outer canopy of eastern- and western-facing rows at a height of 1 m. Derived values of the daily light integral (DLI, in mol photons m^−2^ d^−1^) at the different positions are denoted by arrows.

To better understand the differential effect of the intra-canopy illumination on E- and W-facing plants, we also probed the natural light conditions and photosynthetic activity at the outer parts of the canopy in these plants. PAR was recorded at the E- and W-facing outer canopy and the daily light integral (DLI) was calculated from the recorded values. Note the positioning of the experimental tunnel, with ‘eastern’ plants inclined (~25°) toward the north and ‘western’ plants toward the south (Figure 2). Figure 6 depicts PAR recordings and the derived DLI at the E- and W-facing canopy and above the canopy on two representative sunny days during the winter. On these days, the DLI inside the tunnel, covered by the polyethylene sheet, was 21 and 24 mol photons m^−2^ d^−1^. PAR sensors at the E- and W-facing canopy were positioned such that they mimic light capture by the canopy. The recordings made at the E- and W-facing canopies show that the DLI at the latter was 2.5-fold (January) and 2-fold (February) higher (Figure 6). Higher DLI values, at both sides of the canopy, were recorded in February as compared to January, as expected when days become longer toward the spring. For E-facing plants, the peak in light intensity was around 9:00–9:30, while for W-facing plants, the peak was between 14:00 and 15:00. Furthermore, the light intensity during peak times was much higher for W-facing plants.

**Figure 7:**
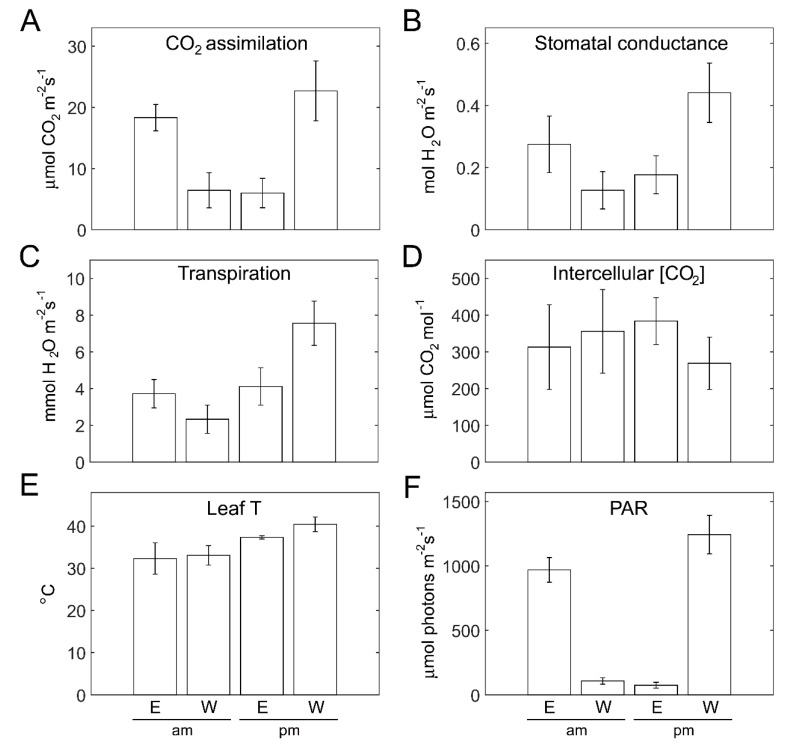
Gas-exchange parameters of the outer canopy of eastern- and western-facing plants in the morning vs. afternoon. (**A**) CO_2_ assimilation rate, (**B**) stomatal conductance, (**C**) transpiration rate, and (**D**) intercellular CO_2_ of leaves of the outer canopy in eastern (E)- and western (W)-facing plants assayed in the morning (am, 8:50–9:30) and afternoon (pm, 13:50–14:30). (**E**) Leaf temperature and (**F**) light intensity (PAR) were recorded during the gas-exchange measurements. Values shown represent means ± SD of 14 leaves (am) and 10 leaves (pm) measured non-destructively.

The photosynthetic activity of plants on the two sides was probed during light peak morning and afternoon hours, using gas exchange measurements of attached outer-canopy leaves (Figure 7). CO_2_ assimilation rates were similar (~6 µmol m^−2^ s^−1^) for the outer canopy side not subjected to direct sunlight: in the morning for W-facing plants and in the afternoon for E-facing plants (Figure 7A). However, the assimilation rates of the W-facing canopy in the afternoon were ~25% higher than those of the E-facing canopy in the morning (Figure 7A). This is due to both the higher light intensity (Figure 7F) and higher leaf temperature (Figure 6E) on the W in the afternoon as compared with E in the morning. Cooling of the canopy via evapotranspiration is prominent for W-facing plants in the afternoon (Figure 7C). The resultant enhanced gas exchange (Figure 7B) contributes toward CO_2_ assimilation in these plants. Lower intercellular CO_2_ is supportive of the higher assimilation in W-facing plants during the afternoon (Figure 7D).

### 3.5. Spring Yield and Plant Biomass

The yield in spring from the experimental sections was summed for six harvests (9 March to 5 May 2020), shown at the top two rows of Table 2. Fruits were picked from the E- and W-facing sides of each section separately and normalized to the number of plants present in each side. For E-facing plants, the yield in LED-D was 30% (kg/plant) and 27% (#/plant) higher as compared to the CR, although these did not pass the significance test. In contrast, the differences of yield in LED-N vs. CR sections for E-facing plants were quite small (~12%). The W-facing spring yield for LED-D was 26% (kg/plant) and 17% (#/plant) higher than CR (not significant). Notably, for LED-N, the W-facing spring yield was 43% higher than the CR both by weight and number of fruits. These results are in agreement with those obtained from the fruit survival quantification (Figure 5B), which show that a significantly higher number of fruits were obtained on the W-facing side.

At the end of the experiment, the plants followed for fruit set and survival were removed and their height and weight were recorded (Table 2). Interestingly, no significant differences were observed for the biomass of E-facing illuminated and CR plants. In contrast, the illumination resulted in considerably heavier and taller plants in the W-facing side. Plants from LED-D were 25% heavier and 12% taller than CR plants, and those from LED-N were 30% heavier and 13% taller than CR. These findings are in line with the fruit set and yield data and support the results of higher assimilation of W-facing plants.

## 4. Discussion

### 4.1. Intra-Canopy Illumination at Passive Conditions

High-cost energy inputs are typically not employed in protected crop cultivation in regions with mostly mild winters, such as the Mediterranean area. However, with the ongoing technological improvements, increasing efficiency of LED lighting, decreasing costs, as well as the potential use of photovoltaic systems as energy sources, commercial application of supplemental illumination at passive conditions can also be envisioned [3,29]. Thus, reports of the use of supplemental illumination in regions previously uncommon are becoming available [13,30].

One of the common applications for high-wire intensive crops is intra-canopy illumination (LED-interlighting) [1], feasible due to the relatively low heat-output of LEDs. Some of the available commercial interlighting LED fixtures provide red (R) and blue (B) light. Improvement of growth, yield, and/or quality using R and B LEDs have been demonstrated for tomato, pepper, and cucumber. R/B interlighting accelerated the ripening of tomato fruit and improved the yield by (+16%) due to increased fruit weight and size [14]. In sweet pepper, interlighting improved the yield and/or quality, with the increase in yield (~16%) arising from a higher number of fruit [10,27]. Recently, R/B interlighting used for mini-cucumber in tropical climate conditions in Brazil resulted in an increased yield (+13% in commercial yield) [13]. In other studies, custom-designed lighting has also been used, with various spectral compositions and ratios of B, R, far-red, and white light applied within the canopy of tomato plants [31,32].

Earlier, we tested several spectral compositions of intra-canopy illumination and found that cool-white (CW) light was preferable for improving the spring yield of winter-grown bell pepper in the Jordan Valley [12]. One evident advantage of using a single type of LED, as opposed to a combination of different wavelength-emitting chips, is a uniform light spectrum applied to the inner canopy. In the current work, we utilized commercial Bioled fixtures providing CW light as intra-canopy illumination for pepper. The photosynthetic and gas-exchange parameters of the inner canopy foliage illuminated with Bioled increased by 3.3- to 5.2-fold, similar to those we observed earlier with other LED fixtures used for bell pepper [12,33]. Using the Bioled product, we extended our previous investigations to testing the application of daytime (LED-D) vs. edge-of-daytime (LED-N) illumination, combined with a detailed characterization of fruit set kinetics during the growth season.

### 4.2. Illumination during Different Times of the Day

While LEDs generate relatively lower heat than other light sources, their use as intra-canopy illumination still inputs heat into the canopy (Figures S1 and S2). At non-controlled growth conditions, it may be disadvantageous to add heat into the canopy during daytime. This is especially true for our area of the Mediterranean, where day temperatures can be quite high. In contrast, low minimal temperatures in the winter may inhibit fruit set. Therefore, it may in fact be more beneficial to illuminate during nighttime or at the edges of daytime, in order to increase the air temperature when they are lowest at night and dawn, while not affecting (increasing) the temperature within the canopy during the hottest hours of the day, at least on sunny days.

The effects of supplemental illumination may differ when provided at different times along the day. Tewolde et al. utilized LED interlighting for supplementing single-truss tomatoes during daytime (4 a.m. to 4 p.m.) or nighttime (10 p.m. to 10 a.m.) [34]. Interestingly, they showed that daytime illumination increased photosynthetic capacity and yield (+27%) only in winter, while nighttime illumination increased photosynthesis and yield in both winter (+24% yield) and summer (+12% yield). Only the winter nighttime illumination was found to be economical in this study [34]. Aside from the plant physiological considerations, night/edge-of-day illumination can also be more cost-effective when powered by electricity, as energy costs may be lower as compared to daytime in some regions [34,35].

With both light regimes applied here, during daytime (LED-D) or edge-of-daytime (LED-N), the fruit set was improved in the winter, with a slight advantage to LED-N. In our conditions and growth season (over winter), bell pepper is characterized by several waves of fruit set during the season. The changing natural light and temperature conditions along the season result in quite different kinetics of fruit development and ripening for fruit set at different times in the growth period. Thus, following the second big wave of fruit set seen in the non-illuminated sections (Figure 4A, Dec. 8), fruits grow and remain on-plant for 2.5 to 3 months. This results in a heavy fruit load, which consequently inhibits additional significant fruit set until the plants are released by the harvest (Figure 4A—black brackets). This is in addition to the prevailing low light and temperature conditions during the period of winter, which may also limit fruit set. Notably, in plants with supplemental illumination, either LED-D or LED-N, higher fruit set occurred in the same time frame when it was quite low in the non-illuminated sections (Figure 4B,C). These results indicate that the added productivity, attained with the supplemental illumination, accounts for the plants’ ability to support additional fruit.

It has been shown earlier that prolonging the photoperiod (using top HPS lamps) in sweet pepper can increase the fruit yield [24,25]. As pepper is a day-neutral plant, the increase in the number of fruits under prolonged days is likely not due to flower induction per se. LED-N exhibited some benefit over LED-D, which may possibly be related to extension of the daily photoperiod in the former. Normally, a fraction of the light-driven photosynthetic assimilates are partitioned toward starch synthesis, utilized by the plant during the dark period. Daytime starch synthesis and its nighttime degradation are highly coordinated to balance the plant metabolic and growth needs, preventing unwanted night starvation responses [36,37]. Modulation of photoperiod length affects starch metabolism, and can therefore affect growth and development [38]; however, the response may differ in different plant species.

When the day length was extended for pepper plants by top lighting, Dorais et al. showed that the daily photosynthate translocation rate increased two- and three-fold for, respectively, photoperiods of 18- and 24 h, compared to 12 h [25]. Under these extended days, fruit yield (kg/plant) increased by 33% (18 h) and 27% (24 h). In our study, the intra-canopy illumination is applied to a fraction of the (inner) canopy, while sunlight affects a different region of the (mostly outer) canopy. Still, the plants illuminated with the LED-N regime are subjected to longer days, of 18 h, as compared to control and LED-D. Prolonging the photoperiod with intra-canopy illumination may have a different effect on the plants than the more conventional overhead illumination, yet both can result in yield increases. The reason that higher yield can be achieved with LED-N than with LED-D may be the availability of sugars from photosynthetic assimilation during the dark hours, and thus alteration of starch metabolism; this direction requires further exploration. The differences between LED-D and LED-N were more pronounced when the two geographical sides of the tunnel were considered, as discussed below. With respect to the idea of the availability of assimilates at night, it would be worthwhile to also test nighttime intra-canopy illumination in our system.

### 4.3. Eastern- vs. Western-Facing Plants

Differences in photosynthesis, growth, and metabolite profiles pertaining to geographical position, reflecting differences in light and temperature conditions, have been documented in grapevine. Assessment of the photosynthetic activity of grapevine leaves at two microsites showed that only east-facing leaves at the (slightly) cooler site were restricted and exhibited lower carbon gain, leading to differential shoot growth [39]. In another study, the diurnal dynamics of the metabolic profile was shown to differ for grape berries positioned toward north-east vs. south-west, implicating that harvest time during the day should be considered [40].

Our findings demonstrate the differential effect of the intra-canopy illumination on eastern- and western-facing plants. Although the illumination was applied symmetrically within the double-row beds (Figure 1A), the effect on W-facing plants was greater. Thus, the fruit set was consistently higher in W-facing plants as compared to E-facing ones of illuminated sections, as compared to the non-illuminated CR sections (Figure 5). Expectedly, the environmental conditions exhibited by the outer canopy differ along the day for E- and W-facing plants. This is exemplified by gas-exchange measurements of outer canopy leaves of E- and W-facing plants during morning and afternoon hours (Figure 7). In the winter, W-facing plants were subjected to higher light intensities in the afternoon hours. This would likely lead to relatively higher canopy temperatures on this side, which, at least for sunny days, persisted for a longer part of the day as compared to E-facing plants. Elevated temperatures can result in higher transpiration and higher stomatal conductance and thus a higher availability of CO_2_, promoting assimilation. We note that vapor pressure deficit (VPD) greatly varies at non-controlled growth conditions, such as the ones in this experiment. Nonetheless, our data suggest that an increased VPD at higher temperatures was not a consistent limiting factor for stomatal conductance and transpiration in W-facing plants. Therefore, it is probable that these plants accumulate more assimilates compared to E-facing ones in winter and early spring. Nonetheless, the fruit set and survival in non-illuminated sections did not differ between the two sides (Figure 5). The differential effect on the two sides was observed only when supplemental illumination is applied. The above indicate that the threshold for supporting additional fruit set and fruit in W-facing plants can be reached earlier, i.e., with less added energy, as compared to E-facing plants. The spring fruit yield and final plant biomass were compared separately for E- and W-facing plants, and significant differences were indeed observed only for W-facing plants. The differences in fruit yield were statistically significant only for LED-N, in line with the higher fruit set observed for this illumination regime.

In conclusion, using cool-white Bioled lighting, we showed that both daytime (LED-D) and edge-of-daytime (LED-N) intra-canopy illumination improved pepper fruit set and fruit survival during the winter at passive conditions. Some additional benefit of LED-N was observed, possibly relating to a longer photoperiod at these conditions. The differential effect of the intra-canopy illumination on eastern- and western-facing plants exemplifies the importance of greenhouse positioning and crop orientation, e.g., the model by [41], and opens additional avenues of investigation for optimizing the use of supplemental illumination under passive growth conditions. These, of course, will likely differ for different crops, as well as for crops grown in different geographical regions of the world.

## Figures and Tables

**Table 1:** Photosynthetic and gas-exchange parameters of the inner canopy ^†^.

Parameter	CR	LED
Chl (µmol m^−2^)	571 ± 59 b	616 ± 57 a
Fv/Fm	0.81 ± 0.01 a	0.81 ± 0.01 a
A (µmol CO_2_ m^−2^ s^−1^)	2.01 ± 0.76 b	6.56 ± 1.87 a
Gs (mol H_2_O m^−2^ s^−1^)	0.030 ± 0.017 b	0.157 ± 0.036 a
E (mmol H_2_O m^−2^ s^−1^)	0.530 ± 0.307 b	1.853 ± 0.264 a
Ci (µmol CO_2_ mol^−1^)	283 ± 48 b	351 ± 32 a
T (°C)	27.7 ± 1.6 a	27.6 ± 0.6 a
PAR (µmol photons m^−2^ s^−1^)	23 ± 6 b	86 ± 36 a

^†^ Measurements were recorded non-destructively on inner canopy leaves from control (CR) and illuminated sections (LED). Recordings were made on leaves found 10–20 cm above the LED fixtures, and at the same height in control sections. For chlorophyll (Chl) and Fv/Fm measurements, n = 15 and 18 leaves, respectively. For gas-exchange measurements, n = 12 leaves from LED-D or CR sections. A, CO_2_ assimilation rate; Gs, stomatal conductance; E, transpiration rate; Ci, intercellular CO_2_. Leaf temperature (T) and light intensity (PAR) were recorded during the gas-exchange measurement. Values shown represent means ± SD; distinct letters denote statistical significant differences (*p* < 0.05) between CR and LED.

**Table 2:** Spring fruit yield and plant biomass.

Parameter	E-Facing			W-Facing		
	CR	LED-D	LED-N	CR	LED-D	LED-N
Yield ^†^ (kg/plant)	1.65 ± 0.37 a	2.14 ± 0.46 a	1.89 ± 0.29 a	1.86 ± 0.25 B	2.34 ± 0.34 AB	2.66 ± 0.48 A
Yield ^†^ (#/plant)	7.74 ± 1.74 a	9.84 ± 2.03 a	8.73 ± 1.51 a	9.12 ± 0.89 B	10.67 ± 1.40 AB	13.05 ± 1.51 A *
Fresh weight ^‡^ (kg)	2.00 ± 0.27 a	2.24 ± 0.54 a	2.24 ± 0.59 a	2.04 ± 0.40 B	2.54 ± 0.69 A	2.65 ± 0.63 A *
Height ^‡^ (m)	2.85 ± 0.32 a	2.89 ± 0.26 a	2.86 ± 0.20 a	2.70 ± 0.30 B	3.03 ± 0.20 A *	3.05 ± 0.23 A *

^†^ Spring yield included six fruit harvests from 9 March 2020 to 5 May 2020 and represent means ± SD from 4 replicate sections. Values were normalized to the number of plants in each side (E, eastern- or W, western-facing) of each section. ^‡^ Plant biomass (fresh weight and height) were measured at the end of the experiment (7 May 2020) on the same plants assayed for fruit set during the season; means ± SD are shown for n = 20 plants (5 plants from the 4 replicate sections of each treatment in E- or W-facing plants). For each parameter, distinct letters denote statistically significant differences (*p* < 0.05) between the three groups facing the same side (lower- or upper-case letters for E- or W-facing, respectively). Asterisks denote *p* < 0.01.

## Data Availability

The data presented in this study are available upon request from the corresponding author.

## Supplementary Materials

The following are available online at www.mdpi.com/article/10.3390/plants11030424/s1, Figure S1: Daily minimal and maximal air temperature within the canopy, Figure S2: Air temperature within the canopy on three representative days, Figure S3: Supplemental intra-canopy illumination improves fruit set in the winter (experiment during 2018–2019), Figure S4: Daily minimal and maximal air temperature.
