# Supplementary material for: Daytime or Edge-of-Daytime Intra-Canopy Illumination Improves the Fruit Set of Bell Pepper at Passive Conditions in the Winter
Source: Plants (Basel). 2022 Feb 4;11(3):424. doi: 10.3390/plants11030424 (PMC8838261; doi:10.3390/plants11030424)
Supplement: Supplementary file 1 [file plants-11-00424-s001.zip › plants-1549255-supplementary.pdf]

Supplemental information

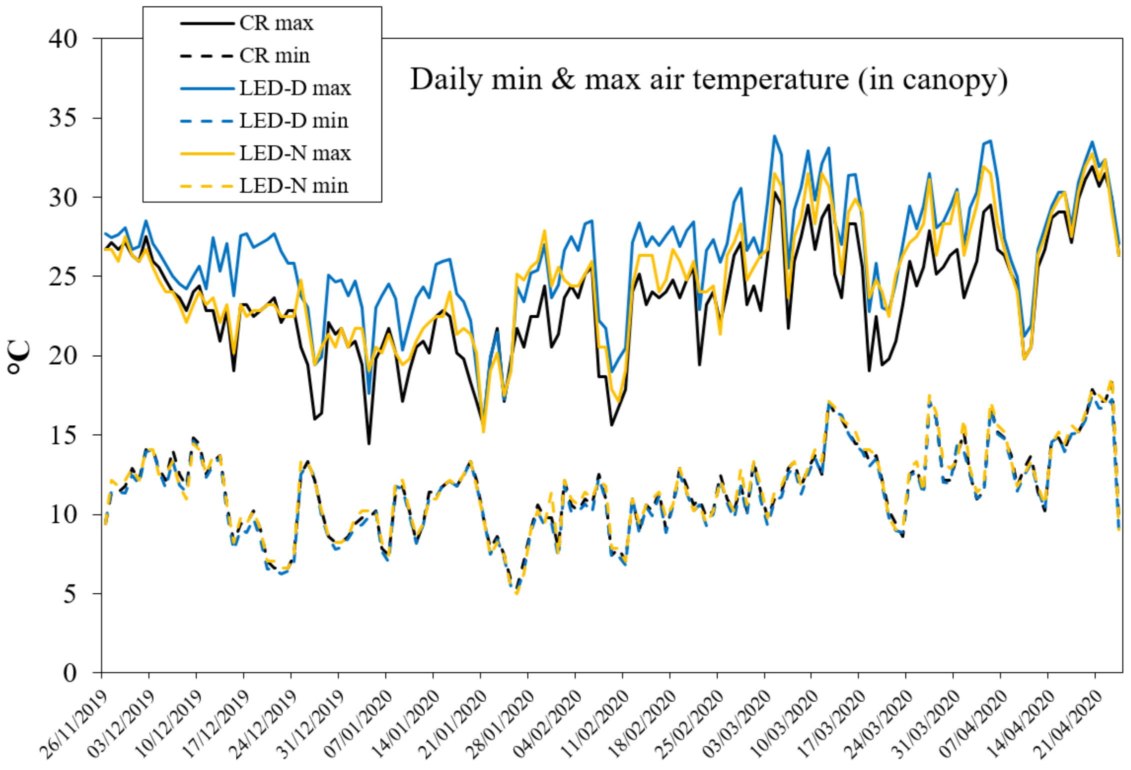

Figure S1. Daily minimal and maximal air temperature within the canopy. Temperature data was recorded using MicroLogs in control (CR) non-illuminated sections, and in sections illuminated during daylight hours (LED-D) and edge of daylight hours (LED-N). Loggers were hung ~15 cm above the LED fixtures, and at the same height in control sections.

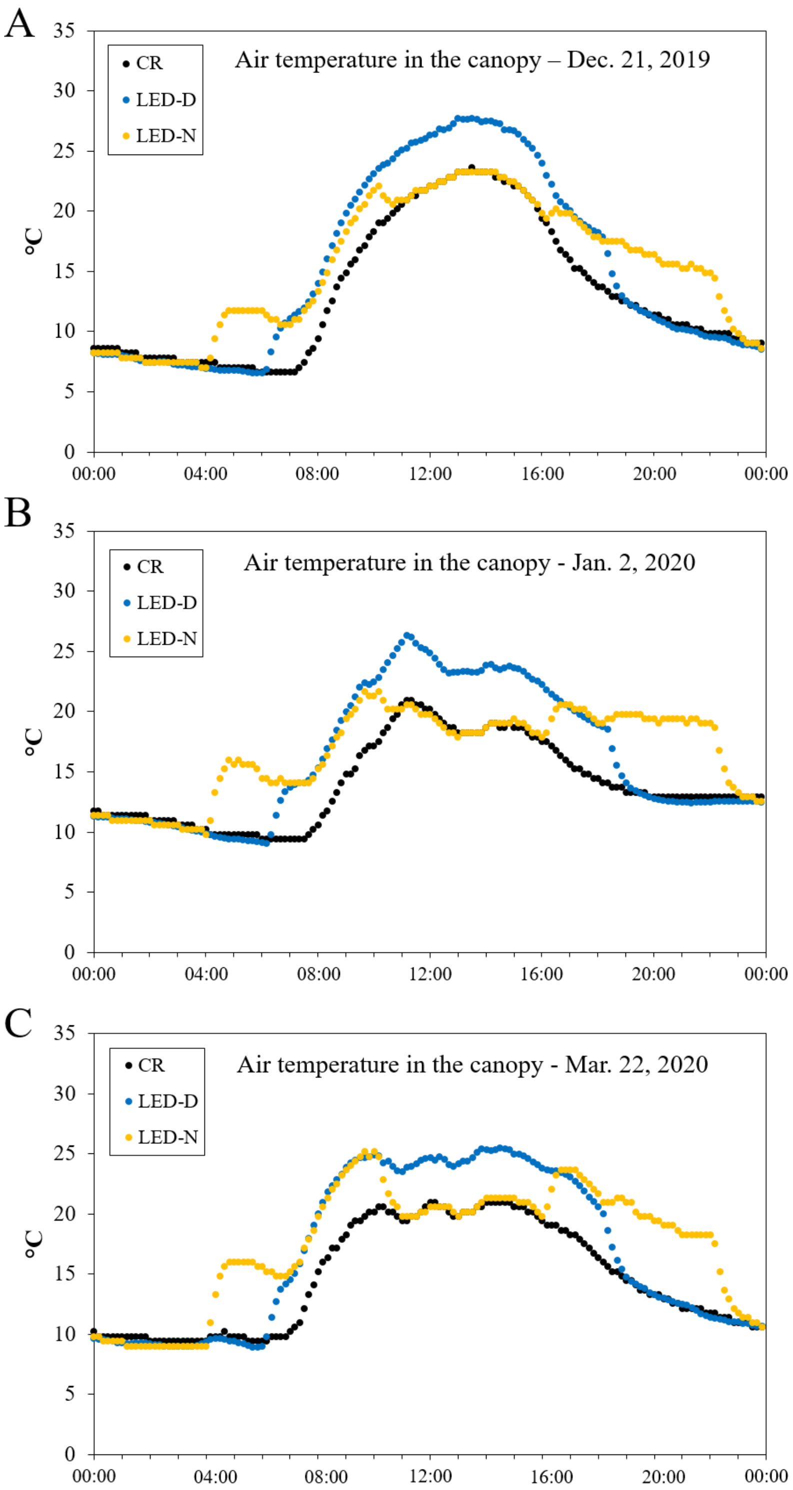

Figure S2. Air temperature within the canopy on three representative days. Raw temperature data, recorded every 10 min. within the canopy of control (CR) non-illuminated sections, and in sections illuminated during daylight hours (LED-D, 06:00-18:00) and edge of daylight hours (LED-N, 04:00-10:00 and 16:00-22:00). Loggers placed as described in Fig. S1.

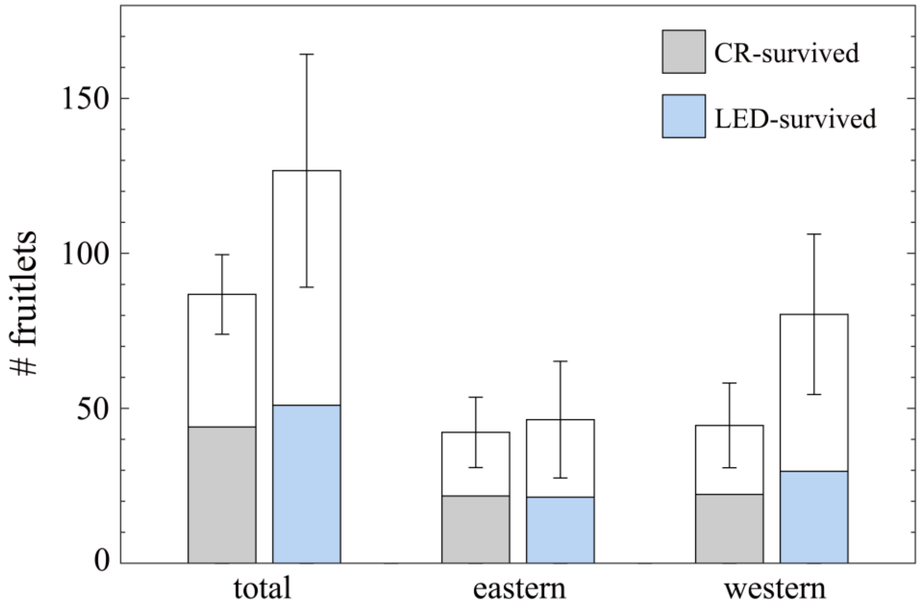

Figure S3. Supplemental intra-canopy illumination improves fruit set in the winter (experiment during 2018-2019). "Total" bars represent the mean cumulative number of fruitlets labelled for a sum of ten plants on three dates in the winter (Jan. 1, Jan. 21 and Feb. 20, 2019) in non-illuminated control (CR) and daytime-illuminated (LED) sections. The total is also divivded down into eastern- and western-facing plants (five plants on each side of the double-row bed; see schematic map and image in main Fig. 1 and Fig. 2A). White parts of the bars denote the aborted fruitlets, while colored parts are the surviving fruit, counted on March 14, 2019. Values shown are means ± SD of three LED sections and four CR sections.

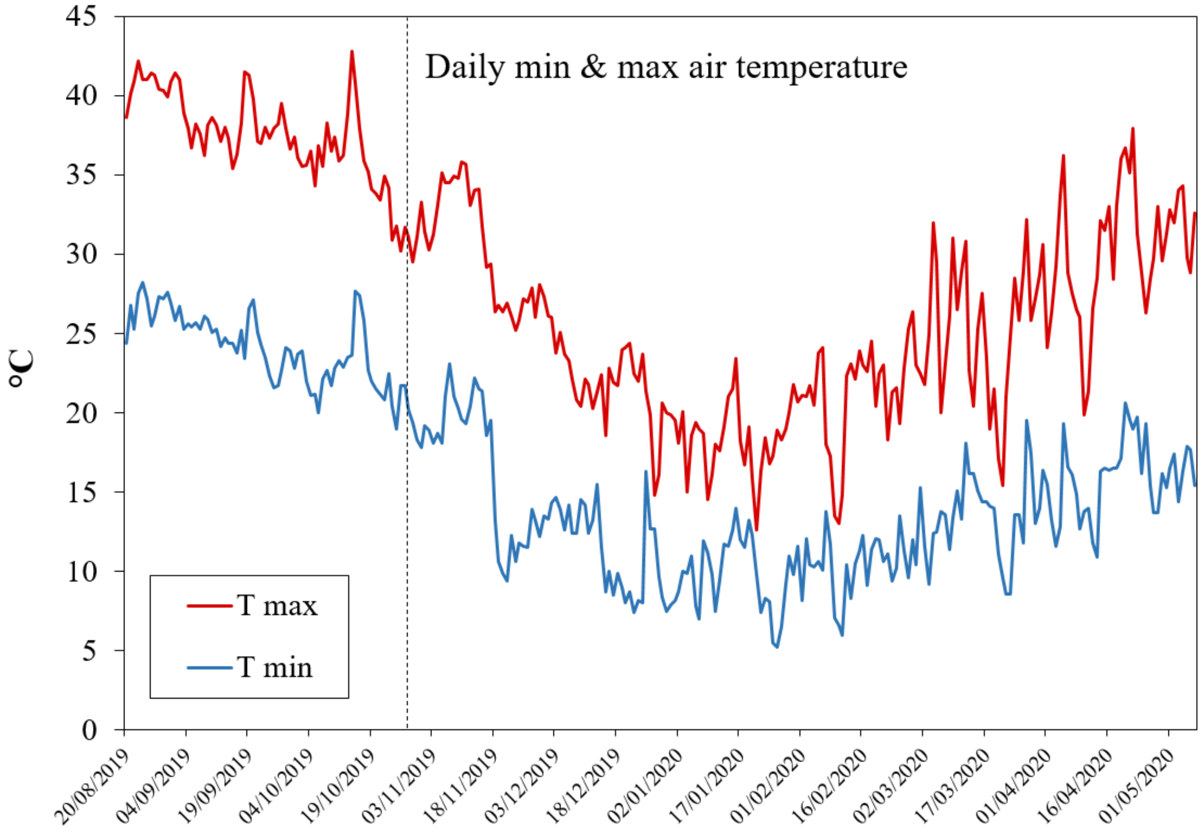

Figure S4. Daily minimal and maximal air temperature. Temperature data (outdoor) was collected at the meteorological station at Gilgal (www.meteo.co.il), shown from date of planting (Aug. 20, 2019) to the end of the experiment (May 7, 2020). Dashed line marks the beginning of illumination period (Oct. 28, 2019).
